# Just Say It Simply: How Prompting Shapes AI Responses for Older Adults

**DOI:** 10.1093/geroni/igaf122.3972

**Published:** 2025-12-31

**Authors:** Litzy Puma, Kalyani Nair, Walter Boot

**Affiliations:** Weill Cornell Medicine, Jamaica, New York, United States; Weill Cornell Medicine, New York City, New York, United States; Weill Cornell Medicine, New York, New York, United States

## Abstract

Large language models (LLMs) have strong potential as decision aids for older adults seeking information related to their health, well-being, and independence. Langston et al. (2025) found that off-the-shelf LLMs, such as Bard (now Gemini) and ChatGPT, provide quick and generally accurate responses to questions relevant to older adults. However, these responses are often long and complex, which may create difficulties due to age-related changes in information processing, particularly for those with mild cognitive impairment (MCI). We tested whether simple prompt modifications, such as stating that the user is an older adult, has MCI, or is seeking a simple explanation, could improve the readability of responses. Three independent raters evaluated a subset responses by ChatGPT4o to questions from Langston et al. (2025), scoring responses on readability metrics. Prompts indicating the user had MCI resulted in slightly simpler language (1.75 vs. 1.80 syllables per word, p < .05), but had little effect on other measures. In contrast, prompts that explicitly requested simple answers led to significantly improved readability, including shorter responses (209 vs. 279 words, p < .05) and fewer syllables per word (1.59 vs. 1.80, p < .001). Sentence length increased (10.87 vs. 9.15 words, p < .001), perhaps because responses contained fewer short filler sentences, more information-dense phrasing, and greater context. These results suggest that prompt engineering can improve LLM usability for older adults, but requests must be direct. LLMs do not reliably adapt based only on descriptions of the user.

